# The eukaryotic initiation factor 5A (eIF5A1), the molecule, mechanisms and recent insights into the pathophysiological roles

**DOI:** 10.1186/s13578-021-00733-y

**Published:** 2021-12-24

**Authors:** Michel Tauc, Marc Cougnon, Romain Carcy, Nicolas Melis, Thierry Hauet, Luc Pellerin, Nicolas Blondeau, Didier F. Pisani

**Affiliations:** 1 LP2M, CNRS, Université Côte d’Azur, Nice, France; 2 Laboratories of Excellence Ion Channel Science and Therapeutics, Nice, France; 3 Service de Réanimation Polyvalente et Service de Réanimation des Urgences Vitales, CHU Nice, Hôpital Pasteur 2, Nice, France; 4 Laboratory of Cellular and Molecular Biology, Center for Cancer Research, National Cancer Institute, Bethesda, MD 20892 USA; 5 INSERM, IRTOMIT, CHU de Poitiers, Université de Poitiers, La Milétrie, Poitiers, France; 6 IPMC, CNRS, Université Côte d’Azur, Valbonne, France; 7 Laboratoire de Physiomédecine Moléculaire, UMR7370, Faculté de Médecine, CNRS, Université Côte d’Azur, 28 Avenue de Valombrose, 06107 Nice Cedex, France

## Abstract

Since the demonstration of its involvement in cell proliferation, the eukaryotic initiation factor 5A (eIF5A) has been studied principally in relation to the development and progression of cancers in which the isoform A2 is mainly expressed. However, an increasing number of studies report that the isoform A1, which is ubiquitously expressed in normal cells, exhibits novel molecular features that reveal its new relationships between cellular functions and organ homeostasis. At a first glance, eIF5A can be regarded, among other things, as a factor implicated in the initiation of translation. Nevertheless, at least three specificities: (1) its extreme conservation between species, including plants, throughout evolution, (2) its very special and unique post-translational modification through the activating-hypusination process, and finally (3) its close relationship with the polyamine pathway, suggest that the role of eIF5A in living beings remains to be uncovered. In fact, and beyond its involvement in facilitating the translation of proteins containing polyproline residues, eIF5A is implicated in various physiological processes including ischemic tolerance, metabolic adaptation, aging, development, and immune cell differentiation. These newly discovered physiological properties open up huge opportunities in the clinic for pathologies such as, for example, the ones in which the oxygen supply is disrupted. In this latter case, organ transplantation, myocardial infarction or stroke are concerned, and the current literature defines eIF5A as a new drug target with a high level of potential benefit for patients with these diseases or injuries. Moreover, the recent use of genomic and transcriptomic association along with metadata studies also revealed the implication of eIF5A in genetic diseases. Thus, this review provides an overview of eIF5A from its molecular mechanism of action to its physiological roles and the clinical possibilities that have been recently reported in the literature.

## Introduction

**Fig. 1:**
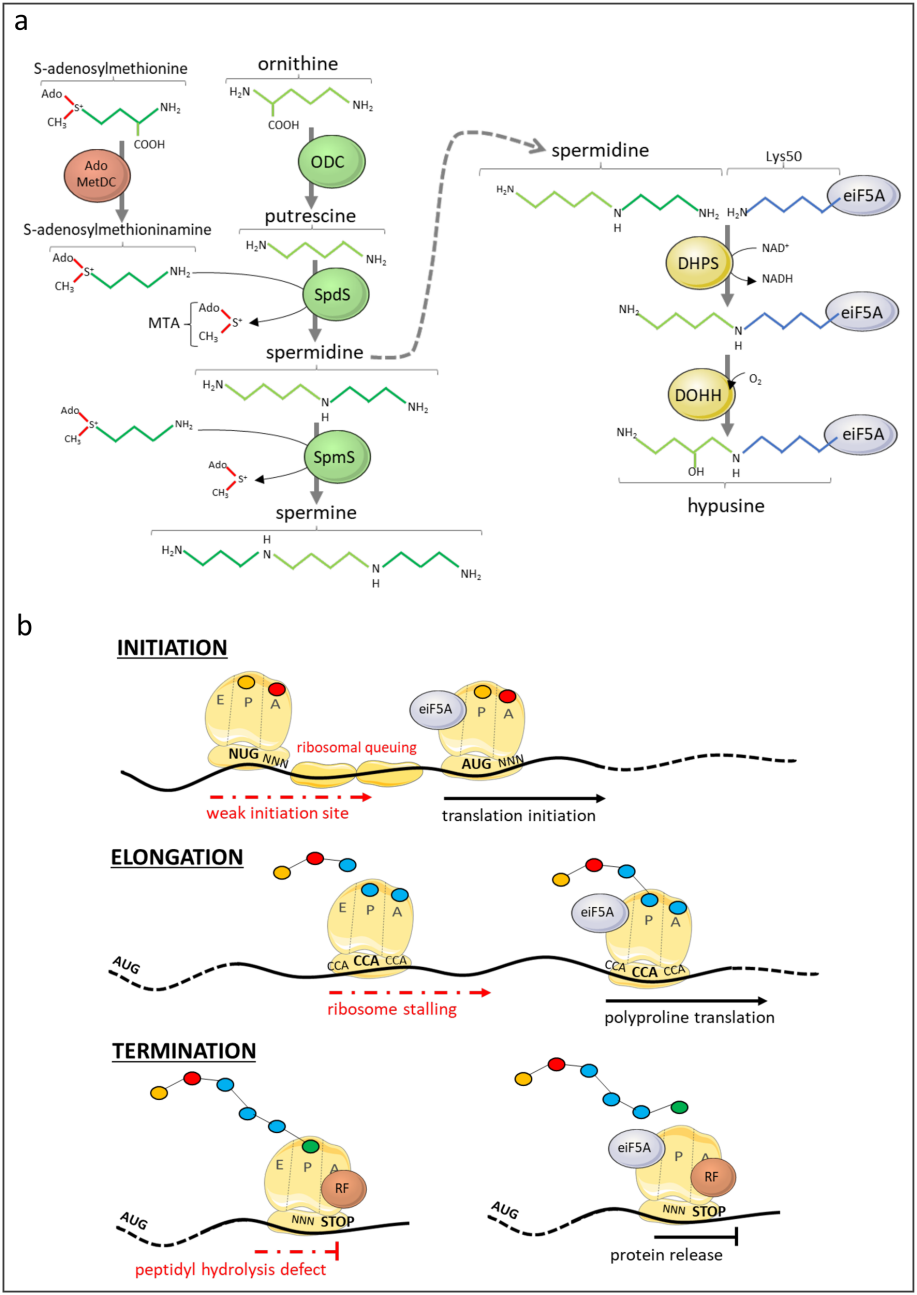
Hypusine modification and role of eIF5A in translation. **a** Polyamine pathway leads to spermidine synthesis which is in turn used by deoxyhypusine synthase (DHPS) and deoxyhypusine hydroxylase (DOHH) to modify Lys50 of eIF5A in hypusine residue. *ODC* ornithine decarboxylase, *SpdS* spermidine synthase, *SpmS* spermine synthase, *AdoMetDC* adenosylmethionine decarboxylase, *MTA* 5′-deoxy-5′-(methylthio)adenosine. **b** Various roles of eIF5A in protein synthesis. At the initiation step, suppression of ribosomal pausing by eIF5A is necessary to maintain the fidelity of start codon selection. Suppression of ribosomal pausing by eIF5A maintains efficient scanning and translation initiation at the appropriate site (right). In the absence of eIF5A paused ribosomes impede scanning, increases dwell time and initiation at upstream leading to non-optimal start codons (left). eIF5A is crucial in elongation step of protein containing polyproline motifs by preserving from ribosome stalling. Finally, eIF5A participates to translation termination at STOP codon by promoting peptidyl hydrolysis due to release factor activity (RF)

In 1976 Kemper et al. [1] purified from rabbit reticulocytes two homogeneous factors named IF-M2Bα and IF-M2Bβ that were initially shown to be required for hemoglobin synthesis. Later on and following the introduction of a uniform nomenclature of initiation factors, it was named eIF5A for Eukaryotic Initiation Factor 5A [2, 3]. eIF5A is a small (17 kDa) acidic protein containing 157 amino acids and is widespread in Archaea and Eukarya. eIF5A is highly conserved along evolution [4] suggesting a vital physiological role as previously shown for embryonic development of mice [5]. It is a relatively abundant protein representing, for example, 1% of the total cellular protein pool of the cytotoxic T lymphocyte [6]. X-ray crystallography data [7] showed that eIF5A1 is composed of two domains with a boundary at residue 83. The N-terminal domain comprises six β-strands and a one-turn 310-helix and contains the hypusine modification site, lysine 50, in the loop connecting β3 and β4, while the C-terminal domain is made up of a three-turn α-helix and five β-strands. eIF5A contains the unusual amino-acid hypusine [8–10] and is unique in that it is the only protein exhibiting this characteristic. Hypusination is a process of post-translational modification of a conserved lysine residue of eIF5A that depends on the presence of spermidine. Spermidine is a polyamine synthesized from putrescine, itself originating from the urea cycle by the catalytic decarboxylation of ornithine. This same spermidine in turn generates spermine. Finally, these three polyamines undergo rapid interconversion in the so-called polyamine pathway [11]. Hypusination of eIF5A occurs through the successive catalytic action of deoxyhypusine synthase (DHPS) and deoxyhypusine hydroxylase (DOHH). In the first step, DHPS catalyzes the NAD^+^-dependent transfer of the 4-amino butyl moiety of spermidine to the ε-amino group of one specific lysine residue (Lys50) of the eIF5A precursor to form a deoxyhypusine residue (7–9). This intermediate is subsequently hydroxylated at carbon 9 by the metalloenzyme DOHH to complete hypusine synthesis and eIF5A maturation (Fig. 1a) [12]. Until now, only the hypusinated form of eIF5A has been reported to have a physiological role. These last few years some important papers report that eIF5A is implicated in various physiological processes including ischemic tolerance, metabolic adaptation, aging, development, and immune cell differentiation. Thus, these newly discovered physiological properties open up huge opportunities in the clinic making the eIF5A pathway an innovative pharmacological pathway. This review is a digest of the main molecular characteristics of eIF5A and focuses mainly on the latest advances regarding its implication in mammalian biology.

## The nature of eIF5A

### Genomic aspect

eIF5A is expressed as two isoforms that share 80% of their cDNA sequences and 94% of their amino acid sequences. The genes encoding eIF5A1 and eIF5A2 are located on different chromosomes. The human *eIF5A1* gene resides on 17p12-p13 and the eIF5A1 protein is ubiquitously expressed in almost all cells and tissues [13], whereas the *eIF5A2* gene resides on chromosome 3q25-q27 and its expression is mainly found in testis, brain, and cancer cell lines and tissues [14–16]. As many publications have focused on eIF5A2 and its relationship with cancer, this review will be mainly dedicated to eIF5A1.

Homo sapiens eIF5A1 has multiple transcripts variants and only two protein isoforms are present. eIF5A1 isoform B (NP_001961.1) encoded by the B and F variants (NM_001370421.1) that contains 154 amino acid residues, and eIF5A1 isoform A (NP_001137232.1) encoded from the A variant with an extension of 30 amino acid residues at its N-terminal end. The expression of the Isoform B predominates over the isoform A as shown in HeLa cells that exhibit a ratio of around 40:1. Isoform B can also be expressed from transcript variant A in a proportion depending on the global sequence environment of the start codons [17]. The biological significance of the additional sequence present in isoform A has been studied and it was proposed that it can direct the protein to the mitochondria [17]. Little is known concerning the expression of the *eIF5A1* gene and its regulation in non-cancerous cells, even if it has been shown to be controlled by p53 the activation of which can up-regulate eIF5A more than ten-fold [18].

### Molecular function

Initially eIF5A was associated to the initiation process of protein translation because it directed the synthesis of methionyl-puromycin [1]. However, it was shown later that depletion of eIF5A in yeast caused only a slight decrease in the overall protein synthesis rate, arguing against a role for eIF5A as a general translation initiation factor. It was shown to be quite a specific factor required for the translation of a subset of certain mRNAs [19, 20]. This hypothesis is supported by the data obtained by Mandal et al. [21], who identified 104 proteins the expression of which was modified in HeLa cells in which eIF5A was deleted through an eIF5A-shRNA-transduced cell strategy. Moreover, increased polysomes in *S. cerevisiae* eIF5A mutants in the absence of functional eIF5A [22] provided evidence that eIF5A is, in fact, involved in the translation elongation process rather than in the initiation one. Moreover, the hypusinated form of eIF5A modifies its capacity to bind ribosomes [23]. In addition to being involved in elongation, eIF5A seems to also be involved in the termination step of protein translation by enhancing the rate of peptidyl-tRNA hydrolysis mediated by eRF1 [24]. In addition, eIF5A is able to rescue polyproline-mediated ribosome stalling as does the bacterial homolog EF-P [25–27]. Using an in vitro yeast reconstituted translation system Abe et al. [28] demonstrated that unmodified eIF5A essentially resolved ribosome stalling; however, the hypusine modification drastically stimulated the ability of eIF5A to rescue polyproline-mediated ribosome stalling and is particularly important for the efficient translation of the N-terminal or long internal polyproline motifs. eIF5A binds to the E-site of the ribosomes [29] and presents the hypusine-containing domain to the P-site where it binds the CCA-end of the tRNA and allosterically allows the formation of a proline-proline bond, to prevent ribosomal stalling on these motifs [30, 31].

Manjunath et al. [32] unraveled the role of eIF5A in the fidelity of start codon selection during the translation process. While studying the growth-promoting gene *MYC* they showed that depletion of eIF5A enhanced upstream translation within 5′ untranslated regions across yeast and human transcriptomes, including on the MYC transcript, which results in increased production of an N-terminally extended protein (Fig. 1b). Thus, eIF5A appeared, beyond its role in suppression of ribosomal pausing, to perform an essential function in maintaining appropriate start codon selection [32]. In the same way, Ivanov et al. [33] identified eIF5A (which competes with polyamines for a common site on the ribosome) as a sensor and effector for polyamine control of uCC translation by analysing the relationships between polyamines, eIF5A, ribosomes and the upstream coding conserved region (uCC) of the antizyme inhibitor 1 (*AZIN1*). Genomic and mutagenic analyzes identified the PPW motif as being crucial for polyamine regulation of uCC and mORF translation, and profiling revealed polyamine-dependent ribosomal pausing on this motif. Interestingly, PPW motifs were the most prominent breakpoints detected in bacteria lacking EFP, a translational elongation factor known to be important for polyproline synthesis. Since the homologous translation factor in eukaryotes, eIF5A, also promotes polyproline synthesis, they showed that eIF5A is necessary for the extension of the PPW motif in uCC and that polyamines act on it. Finally, they proposed that stalling of elongating ribosomes promotes initiation by positioning a ribosome near the start codon.

The N-terminal domain of eIF5A contains the hypusine residue, which carries two positive charges and closely resembles spermidine and spermine which are known to interact with DNA and RNA [34]. Based on this information Xu et al. [35] searched for the physiological RNA targets of eIF5A in HeLa cells and found that eiF5A could also act as a RNA binding protein. They showed that a selected group of mRNAs co-purified with eIF5A either by co-immunoprecipitation or by binding to a His-tagged eIF5A. They identified the AAAGUG consensus sequence able to bind eiF5A and some related proteins. In another study, while looking for the effect of eIF5A in the context of environmental stress Li et al. [36] proposed that eIF5A contributes to the stress granule assembly, that normally helps to reprogram protein expression in a way that promotes cell survival under adverse conditions, for example in RDG3 cells exposed to arsenate. They showed that eIF5A promotes ribosome run-off indicating that it supports translational elongation in cells exposed to adverse environmental conditions.

### Cell distribution

eIF5A was first shown to be localised at the nuclear pore complex where it interacted specifically with the activation domain of the HIV-1 Rev trans-activator protein [37]. Parreiras-e-Silva et al. [38] identified in the N-terminal nucleotide sequence of eukaryotic eIF5A a sequence that may be essential for the localisation of the protein to the nucleus. Later on, Shi et al. [39] described the cytoplasmic localisation of eIF5A. These authors identified two populations of eIF5A in the cytoplasm after obtaining a soluble fraction and a fraction bound to internal membranes in association with the endoplasmic reticulum. The discrepancy in localisation of eIF5A may arise from the use of epitope tagged exogenous eIF5A as a marker for endogenous eIF5A. Indeed, eIF5A exists predominantly in its hypusinated form whereas the FLAG-eIF5A protein remains largely as the non-hypusinated form [40]. It has been observed that several proteins with different biological activities in eukaryotic cells migrate from the cell nucleus into the cytoplasm depending on the energy and signaling through exportins. While studying the effect of hypusination on the intracellular localisation of eIF5A, Lee et al. [40] found that hypusination directs eIF5A into the cytoplasmic compartment and that its nuclear export is mediated by exportin 4 (XPO4) in a hypusine-dependent manner [41]. Due to its small size (17 kD), eIF5A readily leaks through the sieve-like barrier of the nuclear pore complex into nuclei [42]. So, it looses its cytoplasmic function but also might even engage in deleterious off-target interactions, such as nonspecific RNA binding or competition with the ribosome export-adapter Nmd3 [43]. Xpo4 captures such mislocalised nuclear eIF5A and retrieves and relocates it to the cytoplasm [42]. The structure of the corresponding export complex revealed that Xpo4 does not recognize a linear sequence [41]. Instead, intra-repeat loops of Xpo4’s HEAT repeats contact both eIF5A domains, including the essential hypusine, and shield the 25S RNA- and tRNA-binding interface [31, 41, 44]. Therefore, Xpo4 acts like a compartment-specific antagonist of the function of eIF5A and as a suppressor of off-target interactions.

eIF5A has also been shown in yeast to be a cargo for the nuclear export of Pdr6 and participates together with the other cargo Ubc9 to the nuclear/cytoplasm translocation of this biportin [45]. Besides hypusination, eIF5A can also be modified by catalytic acetylation by spermidine/spermine-*N*1-acetyltransferase 1 (SSAT1) at residues K47 and K68 [46]. Ishfaq et al. [47] found that the acetylated form of eIF5A is primarily enriched in the nucleus, whereas unacetylated eIF5A is primarily cytoplasmic and that this distinction is more evident in the presence of histone deacetylase inhibitors. Even if localization of eIF5A depends largely on its post-translational modifications, it can be supposed that it exerts complementary functions in the nucleus and cytoplasm. Indeed, recent advances demonstrated that hypusinated eIF5A is a RNA-binding protein associated to other cargo proteins such as exportins [41, 42, 48], which participate in the nuclear export of specific mRNAs including Nos2 [41, 42, 48], TAR DNA-binding protein 43 (TDP-43) [49] or CD83 [48, 50]. Now, concerning acetylation of the hypusine residue of eIF5A Lee et al. [40] found that acetylation of the hypusine residue by SSAT1 inactivates eIF5A and suggested a potential regulation of the eIF5A activity by reversible acetylation/deacetylation at this site.

## Inhibitors of eIF5A activation

### *N*1-guanyl-1,7-diaminoheptane: GC7

eIF5A is the only protein that is activated by DHPS and DOHH. Thus, inhibiting the activity of eIF5A could be mediated through the specific inhibition of either DHPS or DOHH. Initially, Jakus et al. [51] have concentrated their efforts on the design of inhibitors. They systematically examined several series of compounds structurally related to spermidine as inhibitors of deoxyhypusine synthase and, based on their findings, reported that certain guanidino compounds, which are potent inhibitors in vitro, also effectively inhibit deoxyhypusine synthase in cultured cells. Among these the most effective inhibitor was the *N*1-guanyl-1,7-diaminoheptane (GC7) giving a K*i* value around 10 nM [52], which is 400-fold lower than the *Km* of spermidine. Moreover, co-crystallization data confirmed the specific binding of GC7 to the active site of its biological target [53, 54]. Interestingly 1 µM GC7 inhibited by more than 97% hypusine production in CHO cells whereas the total protein synthesis was only diminished by 10% highlighting the minor role of eIF5A in general protein synthesis. This very selective specificity of GC7 toward DHPS was confirmed later by Lee and Folk [55] who synthetized and tested a number of modified 1,7-diaminoheptanes. The lack of specificity of GC7 and its inability to be used in the clinic due to anticipated side effects is often reported. It should be kept in mind that inhibiting the activation of eIF5A—even with GC7 as the best specific inhibitor up to now for DHPS—will necessarily have various physiological effects because eIF5A directs the expression of different mRNAs. Thus, regardless of the affinity of a DHPS inhibitor, there will inevitably be multiple collateral effects. It has also been reported that GC7, which is a synthetic polyamine, could exert its effects through a global action on the overall cellular polyamines pool. However, one can reasonably consider that the effect of GC7 on this pool is probably negligible due to its K*i* (10 nM) for DHPS as compared to the intracellular polyamines content [56].

### Deoxyspergualin

In 2002 Nishimura et al. [57] analysed the antiproliferative effect of deoxyspergualin, a synthetic derivative of spergualin produced by *Bacillus laterosporus.* Deoxyspergualin inhibits active eIF5A formation and subsequently inhibits cell growth through the inhibition of DHPS. However, the concentration used to obtain this effect was in the millimolar range and needed several days of incubation. These authors proposed that deoxyspergualin may react with amino acid residues located in the active site of DHPS to form covalent linkages. In this case, deoxyspergualin may function as an inhibitor of DHPS after degradation into glyoxyspermidine and guanidinoheptanate amide.

### CNI-1493 (Semapimod, AXD455, CPSI-2364)

While looking for an antiretroviral therapy, and given the fact that eIF5A has been reported to be a cellular cofactor of the Rev pathway involved in HIV-1 replication, Hauber et al. [58] identified the guanylhydrazone CNI-1493 as an efficient inhibitor of human DHPS. They found that the inhibition of DHPS by this agent suppressed the retroviral replication cycle in cultures of cell lines and primary cells. In addition, Schröder et al. [59] reported on the design and the effect of CNI-1493 analogues and found that these derivatives suppressed HIV-1 replication in a dose-dependent manner without toxic side effects. Independently of its action on eIF5A activation CNI-493 was tested in a clinical trial on patients suffering from Crohn’s disease and a clinical response was seen in 67% of the patients at week 4 of treatment [60]. However, in this trial CNI-1493 was used as an inhibitor of mitogen activated protein kinases. It remains to be determined whether these clinical effects were in part related to the inhibitory effect of CNI-493 on eIF5A activation since it later emerged as an inhibitor of DHPS [61].

### DHSI-15

Based on the structure of known DHPS inhibitors Ziegler et al. [62] developed 20 new substances including DHSI-15 which was able to inhibit hypusine synthesis in an in vitro assay. DHSI-15 exerts strong antiproliferative effects on BCR-ABL cells including Imatinib resistant mutants. According to the literature this inhibitor does not appear to have any current use.

### Ciclopirox

The non-heme iron with inside the catalytic center of DOHH renders the enzyme sensitive to small molecule inhibitors that comply with the steric restrictions imposed by the active site pocket and interact with the metal via bidentate coordination [63]. The pharmaceutical ciclopirox (CPX) is a topical antifungal that blocks DOHH activity in the micromolar range and impairs eIF5A maturation by blocking the binding of DOHH to its substrate. In addition, ciclopirox has been shown to inhibit initiation of HIV-1 transcription in which eIF5A plays an important role [63].

### Deferiprone

Deferiprone (3-hydroxy-1,2-dimethylpyridin-4-one [Ferriprox^®^], a 3,4-HOPO), is a systemic iron chelator used by physicians to relieve iron overload in thalassemia and approved by the Food and Drug Administration (FDA) and the European Medicines Agency (EMA). It inhibits the O_2_-utilizing protein hydroxylase DOHH involved in eIF5A hypusination. Deferiprone has been reported to inhibit HIV-1 replication in tissue culture through the blockade of the hydroxylation step of eIF5A activation [64].

### Non spermidine mimetic inhibitors

Very recently Tanaka et al. [53] conducted synthetic studies and obtained bromobenzothiophene, a new compound that targets DHPS with a k*i* around 60 nM. X-ray crystallographic analysis of bromobenzothiophene in complex with DHPS revealed a conformational change in DHPS, suggesting the presence of a novel allosteric site. These findings open the door toward a new clinical approach targeting eIF5A.

### Mimosine

Mimosine [α-amino-β-(3-hydroxy-4-oxo-1,4,dihydropyridine-1-yl)propanoic acid] is a plant amino acid found in the seed and foliage of *Mimosa* and *Leucena*. Mimosine blocks the cell cycle at the G1-S phase interface [65] through the inhibition of the hypusination process. This effect has been attributed to the inhibitory effect of mimosine (as a metal chelator) on DOHH [66]. Mimosine has been shown to have an inhibitory effect on eIF5A hypusination in *Leishmania donovani* [67] and its derivatives in *Plasmodium falciparum* [68] demonstrating that targeting the hypusine pathway as anti-parasitic chemotherapy could be promising.

## Role in cell differentiation

**Fig. 2:**
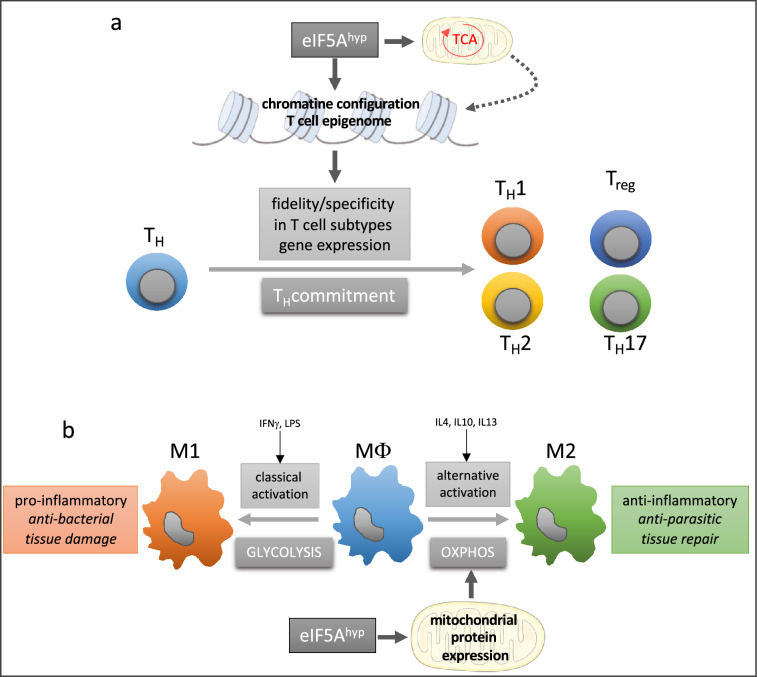
Role of eIF5A in phenotype acquisition of immune cells. **a** Polyamine-hypusine alteration caused epigenetic remodeling due to alterations in histone acetylation and tricarboxylic acid (TCA) cycle rewiring. Thus, polyamine metabolism is essential for maintaining specificity of T cell gene expression to drive T cell subset fidelity. **b** Macrophage polarizations are highly dependent of energy metabolism, glycolysis allowing classical activation of MΦ to M1 pro-inflammatory macrophages and oxidative phosphorylation (OXPHOS) is required to alternative activation in M2 anti-inflammatory macrophages. Hypusination of eIF5A, through control of mitochondrial protein expression, is mandatory for alternative activation of macrophages

The participation of eIF5A in cell proliferation is well established and some authors looked for its participation in embryogenesis and cell differentiation. Parreiras-e-Silva et al. [69] showed that eIF5A is expressed at all mouse embryonic post‐implantation stages with an increase in eIF5A mRNA and protein expression levels and particularly in regions which undergo active differentiation. They corroborated this result with the finding that inhibiting eIF5A in C2C12 cells impairs their differentiation into myotubes and decreases MyoD (a myogenic transcription factor) transcript levels. Another study, using stem cells from rat skeletal muscles revealed that the expression of eIF5A was increased in satellite cells undergoing differentiation in comparison to non‐differentiated satellite cells and skeletal muscle [70]. Moreover, the treatment with GC7 reversibly abolished the differentiation process. In a recent study, Puleston et al. [71] analysed the role of polyamines in the differentiation of CD4+ T_H_ cells into distinct subsets (T_H_1, T_H_2 and T_H_17 cells). They found that cells deficient in ornithine decarboxylase (*Odc1*−/−) exhibited a dysregulation in TH lineage commitment and proliferation. This defect can be restored by putrescine supplementation. Thus, polyamines are essential to CD4+ T cell differentiation. The authors also questioned whether the mechanism by which polyamines control TH cell subset fidelity is through spermidine production and eIF5A hypusination. They showed that synthesis of the amino acid hypusine via the enzyme DHPS underlies the core requirement for polyamine metabolism in directing TH lineage fidelity. They also reported that T cell-specific deletion of the hypusine-synthesizing enzyme DOHH leads to T cell dysregulation and colitis in mice through a dysregulation of the expression of cytokine and transcription factors. They concluded that the polyamine-hypusine axis directs TH lineage commitment by ensuring that the correct chromatin configuration is in place for T cell specification and places polyamine metabolism as a critical regulator of the T cell epigenome (Fig. 2).

## Metabolism

**Fig. 3:**
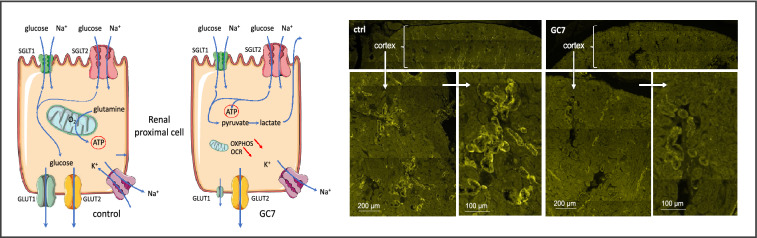
Role of eIF5A in the handling of glucose. Inhibition of eIF5A reprograms metabolism, glucose handling and GLUT1 expression in mouse proximal tubule. **a** Schema of the glucose handling pathway modified by eIF5A inhibition. **b** Immunofluorescence of GLUT1 expression in cortex from control and GC7 treated mouse (Modified from Cougnon et al. [78])

It is known that a deficiency in *dhps* or chronic treatment with DHPS inhibitors, like GC7, enhances glucose tolerance and glycemia in different mouse models of diabetes (HFD [72, 73], STZ [48, 74] humanized mouse model of T1D [75], db/db [76], NOD [77]). Searching for the impact of GC7 mediated eIF5A inhibition on renal glucose metabolism, Cougnon et al. [78] reported that GC7 decreased protein expression of the renal GLUT1 glucose transporter in cultured proximal cells resulting in a decrease in trans-cellular glucose flux. In parallel, GC7 modified the native energy supply of the proximal cells from glutamine toward glucose use. Thus, GC7 acutely and reversibly reprograms the function and metabolism of kidney cells to make glucose its single substrate, and therefore permits cells to be oxygen-independent through anaerobic glycolysis. The physiological consequences in GC7-treated mice [78] are an increase in the renal excretion of glucose and lactate reflecting a decrease in glucose reabsorption and an increased glycolysis, a phenomenon also reported in cultured proximal cells [79]. How eIF5A can repress GLUT1 expression remains to be determined since the GLUT1 sequence does not contains any polyproline motif and its mRNA sequence does not harbor the consensus sequence required to link eIF5A (Fig. 3).

Using yeasts that expressed the two yeast eIF5A analogs Tif51A and Tif51B Barba-aliaga et al. [80] analysed how eIF5A expression is regulated to adapt yeast to the metabolic requirements. They observed that only the isoform Tif51A and not Tif51B, is needed for growth in the presence of glycerol and ethanol as carbon sources. Under those conditions, the isoform TIF51A was up-regulated and the oxygen consumption was reduced by TIF51A depletion. Complete TIF51A hypusination was not needed to maintain high levels of respiration. The authors concluded that the yeast Tif51A isoform responds to the metabolic state of cells to promote mitochondrial respiration.

## Apoptosis and mitochondria

The relationship between eIF5A and apoptosis has been reported in cancer cell lines [23, 81, 82], however the mechanisms underlying this link remains somehow controversial. It appears that the role of hypusinated eIF5A depends on the stress that the cells have to face. Thus, for example, GC7 has been shown to have a synergistic effect on apoptosis together with the apoptotic inducer IFNalpha [83]. Conversely, it exerts an anti-apoptotic effect in renal cell submitted to anoxia [79] through a metabolic shift toward anaerobic glycolysis and a mitochondrial remodelling characterized by a drop in the expression of OXPHOS mitochondrial complexes. This modulation of OXPHOS by hypusinated eIF5A has also been reported by Puleston et al. [84] in macrophages in which GC7 also dampens the expression of mitochondrial proteins. This anti-apoptotic effect has also been reported in HT29 cells, transfected with an eIF5A siRNA, and exposed to the apoptotic inducer sodium nitroprusside, a classical nitric oxide donor [85]. In the same study the authors reported that over‐expression of eIF5A1 induced activation of caspase-3, -8, and -9 together with a loss of the mitochondrial transmembrane potential, release of cytochrome *c*, and translocation of Bax. Analysing the mitochondrial dynamics linked to cellular senescence Ma et al. [86] reported that the Kruppel like factor 5 (KLF5 an essential transcriptional factor of cardiovascular remodeling, that mediates the link between mitochondrial dynamics and vascular smooth muscle cell senescence) knockdown enhanced, while KLF5 over-expression suppressed mitochondrial fission. Mechanistically, KLF5 activates eIF5A transcription through binding to its promoter, which in turn preserves mitochondrial integrity by interacting with mitofusin 1 (Mfn1). On the other hand, decreased expression of eIF5A promoted by KLF5 down-regulation leads to mitochondrial fission, reactive oxygen species (ROS) production and apoptosis. The involvement of eIF5A in triggering apoptosis has also been reported by Miyake et al. [87] in ovarian cancer cells through the inhibition of the nuclear export system. These authors reported that IGF2BP1 (a member of the RNA-binding IGF2BP family) binds to eIF5A in the cytoplasm and that inhibition of nuclear export of IGF2BP1, mediated by exportin 1 (XPO1/CRM1) [88], decreases the capacity of IGF2BP1 to bind eIF5A. In IGF2BP1-depleted cells, eIF5A accumulates in the mitochondria and apoptosis is induced. These findings would mean that IGF2BP1 works as an eIF5A binding protein and regulates the function of eIF5A in apoptosis.

eIF5A is predominantly present as the isoform B and contains only 154 amino acids. The isoform A has an extended N‐terminal sequence containing 30 amino acids not present in the isoform B and drives its association with mitochondria [17]. Thus, the question is whether this interaction has some functional relevance, even if the isoform A is quantitatively lower compared to the isoform B. Pereira et al. [89] reported that depletion of the isoform A, using siRNA specific to the EIF5A1 variant A (siVA), modulated oxidative metabolism in association with down-regulation of mitochondrial biogenesis‐related genes. They observed a decrease in the protein content of complexes II and V and an increase in complex III, in parallel with a metabolic shift from oxidative to glycolytic metabolism. The same result has also been obtained in renal cells treated with GC7 that impaired eIF5A hypusination of both isoforms [78, 79]. However, cells treated with siVA show a significant increase in respiration coupled to oxidative phosphorylation. The addition of a mitochondrial uncoupling protonophore showed that the maximal respiratory capacity was significantly higher in siVA treated cells than in the siControl. The opposite occurs in renal cells in which GC7 reduced drastically the basal oxygen consumption rate indicating a decrease in the mitochondrial oxidative phosphorylation and an enhancement in anaerobic glycolysis [78, 79]. This reversible GC7 induced mitochondrial “silencing” is accompanied by a non-lethal decrease in the mitochondrial potential [78, 79]. Puleston et al. [84] reported the same results in macrophages in which GC7 leads to an inhibition of several mitochondrial proteins explaining the observed shift to anaerobic glycolysis. It remains to be established whether complete suppression of eIF5A expression has the same effect as inhibition of its hypusination step alone and whether non-hypusinated eIF5A has a not yet elucidated physiological role.

Naturally, inhibition of eIF5A modifies the metabolic status by down-regulating mitochondria with a direct impact on the abundance of various metabolites originating from the tricarboxylic acid cycle, metabolites that have been shown to be involved in the control of cellular function and fate in different contexts [90]. This is important when considering the new concept [91] of chromatin remodelling depending, among others aspects, on the level of electrophilic metabolites produced in pathways such as acyl-CoA synthesis [92], lipid degradation [93], and glycolysis [94]. Since eIF5A that is potentially able to modify the level of these metabolites together with the level of polyamines one can ask whether it is involved in epigenetic phenomena such as the one involved in the helper T cell lineage fidelity [71].

## Anti-microbial response

Polyamine metabolism plays a crucial role in bacterial infections [95], including those of the gastrointestinal tract in which macrophages respond to foreign antigens and develop strategies to clear pathogens [96]. Gobert et al. [95] hypothesized that protein translation through the eIF5A pathway is crucial for the adequate response of macrophages to invaders and found that myeloid cell-specific deletion of *Dhps* in mice infected with *Helicobacter pylori* (*H. pylori*) and *Citrobacter rodentium* leads to reduced eIF5A hypusination in macrophages and increased bacterial burden and inflammation. In response to *H. pylori* infection mice bone-marrow-derived macrophages exhibited a significant increase in *Dhps* mRNA expression whereas the *Dohh* mRNA level remained unchanged. Moreover, gastric biopsies from uninfected individuals and *H. pylori*-infected patients with gastritis revealed that the number of cells with double staining for both hypusinated eIF5A and the macrophage marker CD68 was significantly increased in tissues from infected patients compared with those without infection. Analysing the proteome of RAW 264.7 (a murine macrophage cell line) treated with GC7 prior to infection with *H. pylori* revealed that, among other proteins, NOS2 was downregulated leading to a decline in NO_2_^−^, the stable metabolite of NO derived from NOS2 activity. In conclusion, in a more general way, the immune pathways involved in the activation and anti-microbial effects of macrophages were down-regulated by GC7 in *H. pylori*-infected RAW 264.7 macrophages. Moreover, it is noteworthy that pathways predicted to have an impact on cell death, including by autophagy, were also negatively affected by the inhibition of hypusination [95]. A role for the hypusinated form of eIF5A in regulating differential macrophage activation has also been delineated. Hypusinated eIF5A was not increased in bone-marrow-derived macrophages following exposure to LPS/IFN-γ, while it was induced after activation with IL-4, demonstrating that hypusinated eIF5A levels are differentially modulated according to the type of immune stimuli [84]. This has to be related to the fact that IL-4-driven activation of these macrophages depends on mitochondrial respiration, while lipopolysaccharide (LPS)/interferon γ (IFN-γ)-driven activation depends on aerobic glycolysis.

## Anti-retroviral therapy

Polyamines and hypusinated eIF5A are involved in the replication of numerous viruses; however, their role in supporting viral replication remains unclear. Viral multiplication requires multiple intracellular interactions of both viral components and host cell factors. The HIV-1 Rev protein is essential for viral replication because it is required for the nucleo-cytoplasmic transport of HIV-1 mRNAs containing the Rev response element (RRE) and hypusinated eIF5A was shown to be a cellular partner of the HIV-1 Rev protein [37] through its ability to bind the nuclear export signal of Rev. eIF5A depletion, anti-eIF5A antibodies, or expression of eIF5A mutants blocks the nuclear export of the Rev protein and HIV-1 replication [97]. Inactive eIF5A mutants that retain the ability to bind Rev impair the export of Rev–CRM1 complexes to the cytoplasm [37, 98] and T CD4 lymphocytic cell lines over-expressing these mutants failed to sustain the efficacy of HIV-1 replication [99]. However, the assumption that eIF5A binds the Rev NES was challenged by Henderson and Percipalle who showed that purified eiF5A did not bind neither to the Rev Nuclear Export Signal (NES) nor to Rev-Response Element (RRE) complexes [100]. HIV-1 blocks programmed cell death, an innate mechanism of defense against infective agent invasion. Using ciclopirox and deferiprone Hanauske-Abel et al. [101] also showed that these compounds were able to activate programmed cell death preferentially in HIV-1-infected cells. The two drugs enhanced mitochondrial membrane depolarization, initiating the intrinsic pathway of apoptosis to execution, as found by caspase-3 activation, poly(ADP-ribose) polymerase proteolysis, DNA degradation, and an apoptotic cell morphology. These authors hypothesized that the mechanism is initiated by the inhibition of DOHH mediated-eIF5A hydroxylation and that targeting this pathway may provide a true pro-apoptotic medical aid against HIV-1 infection. This idea was applied to a clinical trial (NCT02191657) [102] in which deferiprone, through the inhibition of DOHH, releases the innate apoptotic defense of HIV-infected cells from infective agent blockade, therefore depleting the cellular reservoir of HIV-1 DNA. It remains to be determined whether the use of a more specific inhibitor such as GC7 is also active against viral invasion.

The Ebola virus (*EBOV*) is one of the foremost deadly pathogens known. It is currently acknowledged, due to its restricted ordering in size, that its replication depends for the most part on host proteins and molecules [103]. Olsen et al. [104] demonstrated that polyamines and hypusinated eIF5A are both required for *EBOV* gene expression. Indeed, blocking the polyamine pathway with DFMO impairs viral transcription by a loss in the mRNA accumulation whereas blocking the hypusination of eIF5A with GC7 impairs the translation of *EBOV* mRNAs. Moreover, data suggested that the dependence on eIF5A was due to the need for hypusinated eIF5A for the accumulation of the VP30 protein, which is a viral polymerase cofactor essential for *EBOV* gene transcription [105]. Mechanistic insight into the processes mediated by eIF5A and implicated in *EBOV* replication deserves to be further studied and will contribute to the development of therapeutic strategies to fight against infection. Because of its role in mRNA shuttling between the nucleus and the ribosome machinery it will be interesting to analyse further the impact of eIF5A on viral replication. Cáceres et al. [106] reported that inhibition of the DOHH activity by specific drugs or a decrease in the DOHH concentration in cells hampers the activity of at least three different retroviral IRESs confirming that IRES-mediated initiation of translation of retroviral mRNAs can be envisaged as a target for the future development of novel antiviral therapies.

## Anti-parasitic therapy

Parasitic invasion is responsible for human diseases and for millions of deaths annually worldwide. Since eIF5A is highly conserved and is essential to all eukaryotes it may represent a new option for a pharmacological targeting of various diseases due to eukaryotic pathogens. A recent study by Jeelani and Nozaki [107] reported that eIF5A and its post-translational modification is essential for both proliferation and differentiation of *Entamoeba histolytica*, the protozoan responsible for amoebic dysentery and liver abscess in humans. They showed that Entamoeba eIF5A is involved in excystation, conversion from the cyst to the trophozoite, but not in encystation.

Nguyen et al. [108] reported that eIF5A and the modification of deoxyhypusine are essential for another protozoan parasite, *Trypanosoma brucei* (*T. brucei*), the causative agent of human trypanosomiasis and are required for adequate expression of proteins containing poly(Pro) residues. Analysis of the expression of two representative essential proteins of *T. brucei* containing nine consecutive prolines (formin and CAP/Srv2p) showed that expression was drastically reduced after knock-down of eIF5A whereas their mRNA levels increased.

The parasite *Plasmodium falciparum* (*plasmodium f.*) possesses genes for both hypusination enzymes, which are supposed to be potential targets of antimalarial drugs. Aroonsri et al. [109] investigated the function of *plasmodium f.* DHPS [110] function in transgenic *P. falciparum* parasites. They found that alterations in its expression result in defects in hypusination of eIF5A in growth of transgenic parasites. Although GC7 targets *plasmodium f.* DHPS with a lower efficacy than in mammals, the inference that *plasmodium f.* DHPS as the primary antimalarial target of GC7 is supported by in-silico modeling data, which show conservation of the substrate binding pocket and *plasmodium f.* DHPS residues putatively involved in interaction with GC7 [109]. Other inhibitors of eIF5A hypusination have been tested with some success as antiparasitic drugs, including derivatives of mimosine and cyclopyroxamine [68] and the guanylhydrazone CNI-1493 [111].

*Leishmania donovani* (*L. donovani*) is a protozoan parasite that causes visceral leishmaniasis. Chawla et al. [67] have recently reported two DHPS-like genes in *L. donovani* that show a low degree of homology with human DHPS. Both genes were cloned and expressed, but only one, DHPS34, exhibited deoxyhypusine synthase activity. Gene replacement studies for DHPS34 indicated that the enzyme deoxyhypusine synthase and eIF5A modification play an essential role in the cellular viability of this pathogenic organism. Despite conservation of some of the active site amino acid residues between the human and leishmanial DHPS, GC7 inhibited only slightly *L. donovani* proliferation. This suggests a topological difference in the spermidine binding sites between the human and the leishmanial enzymes and opens up the possibility that the differences between the two enzymes could be exploited for drug development for visceral leishmaniasis [67, 112]. The importance of eIF5A in the fight against *Leishmania* has also been reported by Duarte et al. [113] who engineered a polyproteins vaccine raised against eIF5A and another hypothetical protein named LbHyp cloned from *Leishmania braziliensis*. Mice immunized with this vaccine were then challenged with either *L. amazonensis* or *L. infantum* promastigotes. The vaccinated animals showed a significant reduction in the number of parasites in all organs and protection was attributed to CD4+ and CD8+ cells that mediate the IFN-γ production against parasites [113, 114].

## Pathophysiological role in major human diseases

Inhibition of eIF5A activation leads to an anti-proliferative effect [12, 115] mediated by arrest of cell cycle progression at the G1-S boundary [66]. This characteristic has stimulated considerable attention from a therapeutic perspective for various types of cancer [116, 117] in which the paralog eIF5A2 is strongly expressed [118]. The role of eIF5A2 in pathogenesis has been recently reviewed by Ning et al. [117] and numerous clinical trials using difluoromethylornithine (DFMO) have been reported [119, 120]. Currently the Food and Drug Administration (FDA) approves DFMO for female hirsutism, human African trypanosomiasis or sleeping sickness [121]. However, DFMO is also an irreversible suicide inhibitor of ODC, the first and rate-limiting enzyme of polyamine biosynthesis, leading in fine to eIF5A inhibition due to the DFMO induced lack of spermidine [84, 122, 123].

## Diabetes and pancreas

**Fig. 4:**
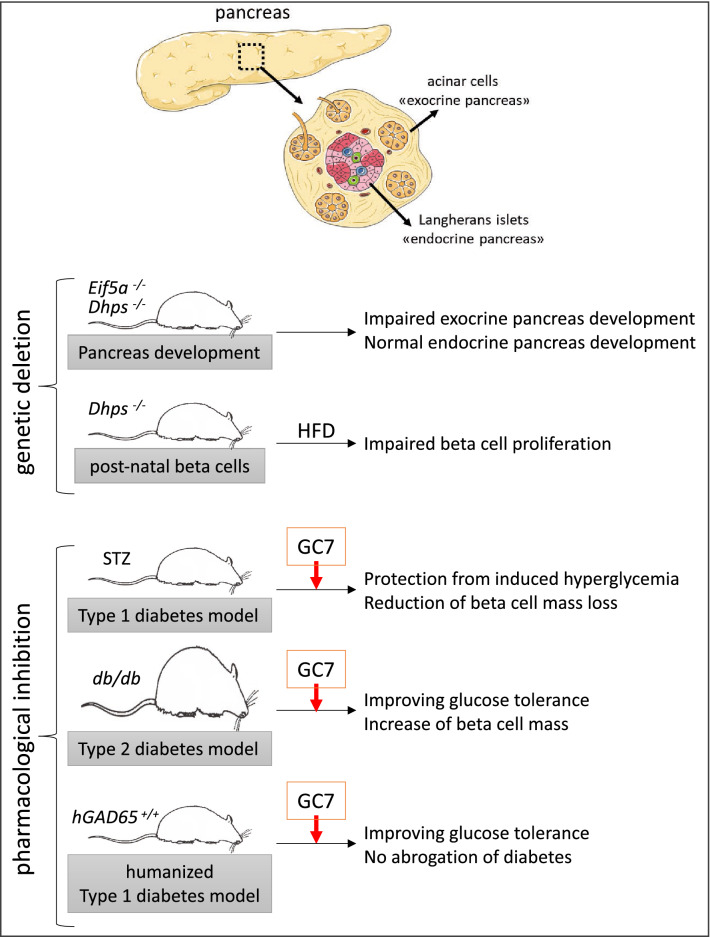
Role of eIF5A in normal and pathologic development of pancreas. Genetic deletion (upper panel). Developmental impact of *Dhps* and *eIF5A* knock-out specifically in pancreas of mice (from Padgett et al. [129]). Inducible knock-out of *Dhps* in pancreatic beta cells following by high fat diet inducing hyperglycemia (from Levasseur et al. [72]). Pharmacological inhibition (lower panel). Impact of GC7 treatment (DHPS inhibitor) on pathological features of Type 1 (streptozotocin “STZ” treatment, from Maier et al. [48]) or Type 2 diabetes (db/db mice mutated for Leptin receptor, from Robbins et al. [76]) mouse models. Impact of GC7 treatment on humanized (antigen presenting cells expressing hMHCII) transgenic mice for hGAD65 (glutamic acid decarboxylase) immunized with adenoviral vectors carrying GAD65, leading to auto-immune response against beta cells and ultimately to Type 1 diabetes induction (from Imam et al. [75])

Diabetes is a disease involving glucose homeostasis related to the dysfunction of pancreatic beta cells. Type 1 diabetes is the result of autoimmune destruction of beta cell islets and type 2 diabetes is thought to develop because the release of insulin from beta cells cannot meet the need for insulin [124]. Pro-inflammatory cytokines from immune cells play an important role in activating signalling pathways that lead to beta cell dysfunction. Since hypusinated eIF5A reduced the level of inflammatory cytokines in a sepsis mouse model [125] its potential involvement in diabetic diseases was studied. In a mouse model of streptozotocin-induced diabetes Maier et al. [48] reported that GC7 was able to protect mice from induced hyperglycemia by reducing beta cell mass loss. They showed that eIF5A is involved in NOS2 production in pancreatic islets and that depletion of eIF5A protects them from streptozotocin toxicity by inhibiting the nucleo-cytoplasmic transport and the translation of the mRNA encoding NOS2 in primary pancreatic islets. Interestingly, *dhps* haploinsufficiency is sufficient to decrease NOS2 expression [126]. Finally, they concluded that cytokine receptor signalling triggers the nuclear translocation of NF-κB, which activates transcription of the *Nos2* gene. *Nos2* mRNA are shuttled out of the nucleus in a CRM1- and eIF5A-dependent manner, and then delivered to ribosomes, where translation occurs to form NOS2. p38 MAPK could participate in this process through a permissive effect on the hypusination rate of eIF5A [127]. Nitric oxide produced by NOS2 suppresses the production of ATP and ultimately inhibits the release of insulin. In this model, eIF5A in its hypusinated form, binds specifically to *Nos2* mRNA. Inhibition of eIF5A by GC7 impairs the nuclear membrane transport of these transcripts that is mediated in an exportin1/CRM1(XPO1)-eIF5A-dependent fashion. The physiological result of GC7 use is improved glucose tolerance [76, 77]. In a more general way, cytokines also stimulate cross-pathways including ER stress in the case of pancreatic islet inflammation. The transcription factor C/EBP homologous protein (CHOP) is a major actor in the perturbation of the endoplasmic reticulum by triggering the unfolding response that leads to cell apoptosis. Robbins et al. [76] found that in obese diabetic C57BLKS/J-db/db mice treatment with GC7 improved glucose tolerance, increased insulin release, and increased the beta cell mass (Fig. 4). This result correlated with a blockade of CHOP protein production despite 30-fold activation of the *Chop* gene. Blocking CHOP translation led to a reduced downstream caspase-3 cleavage and a protection of the cells from apoptotic death. Despite these data, and using a “humanized” transgenic mouse model for type 1 diabetes, Imam et al. [128] failed to demonstrate a protective effect of inhibition of DHPS on the development of type 1 diabetes, even if down-regulation of eIF5A changed the pathophysiology, and observed immune outcome of diabetes in an animal model that is very similar to that of human type 1 diabetes (Fig. 4).

In addition to the role of eIF5A in the diabetes-induced inflammatory response, it has been shown to play a critical role in the development of the pancreas. Using conditional allele approaches to decipher and put forward the requirement of hypusine in this process, Padgett et al. [129] showed that genetic deletion of *Dhps* in the pancreas leads to altered mRNA translation and reduced protein synthesis, including a specific decrease in the synthesis of digestive enzymes and proteins, including lipase, elastase, carboxypeptidase A, and amylase, that influence acinar cell development of the pancreas (Fig. 4). Moreover, these authors confirmed that deletion of *Dhps* and *Eif5a* is not required for endocrine cell development in the pancreas.

Looking for a possible effect of DHPS on the beta cell mass throughout the development of diabetes, Levasseur et al. [72] generated a tissue-specific inducible deletion of *dhps* in pancreatic islets of mice. When fed a high fat diet (HFD) these mice developed diabetes with a lower level of glucose tolerance. In addition, unlike the HFD-fed controls, these mice were unable to gain beta cell mass (an indicator of obesity-induced diabetes) and this was due to a lack of cell proliferation rather than increased cell death. The cell proliferative phenotype in *dhps*-KO mice was attributed to a decrease in cyclin D2 expression, while the coding *Ccnd2* mRNA (key mRNA which is crucial for beta cell proliferation) remained unchanged. The authors concluded that these results suggest a reduced translational initiation of *Ccnd2* in DHPS deficient mice pancreatic islets. In addition, the pro-proliferative effect of c-Myc (a key regulator of cell proliferation) in mouse and human pancreatic islets is impaired when DHPS is inhibited. Finally, eIF5A hypusination links increased insulin demand caused by insulin resistance with beta cell proliferation to maintain glucose homeostasis [72]. These data obtained from different teams revealed that opposite results could be obtained if we consider a genetic deletion of eIF5A versus a pharmacological inhibition using GC7. In the first case beta cell proliferation is impaired while in the second the beta cell mass is increased together with an improvement in glucose tolerance (Fig. 4).

Pro-inflammatory macrophage infiltration into adipose tissue is a hallmark of the metabolic inflammation of obesity that leads in fine to diabetes. Anderson-Baucum et al. [130] found that the hypusinated form of eIF5A was increased in adipose tissue macrophages M1, which exhibit a proinflammatory phenotype in obese mice. They report that targeting DHPS in myeloid cells of obese mice decreased M1 macrophage infiltration and improved the sensitivity to insulin. They concluded that DHPS, whose only role known so far is the activation of eIF5A, contributes to a pro-inflammatory M1-like phenotype. These data suggest that targeting the pair DHPS/eIF5A could serve to modulate macrophage activation to restore glucose tolerance.

## Brain aging and neural development

**Fig. 5:**
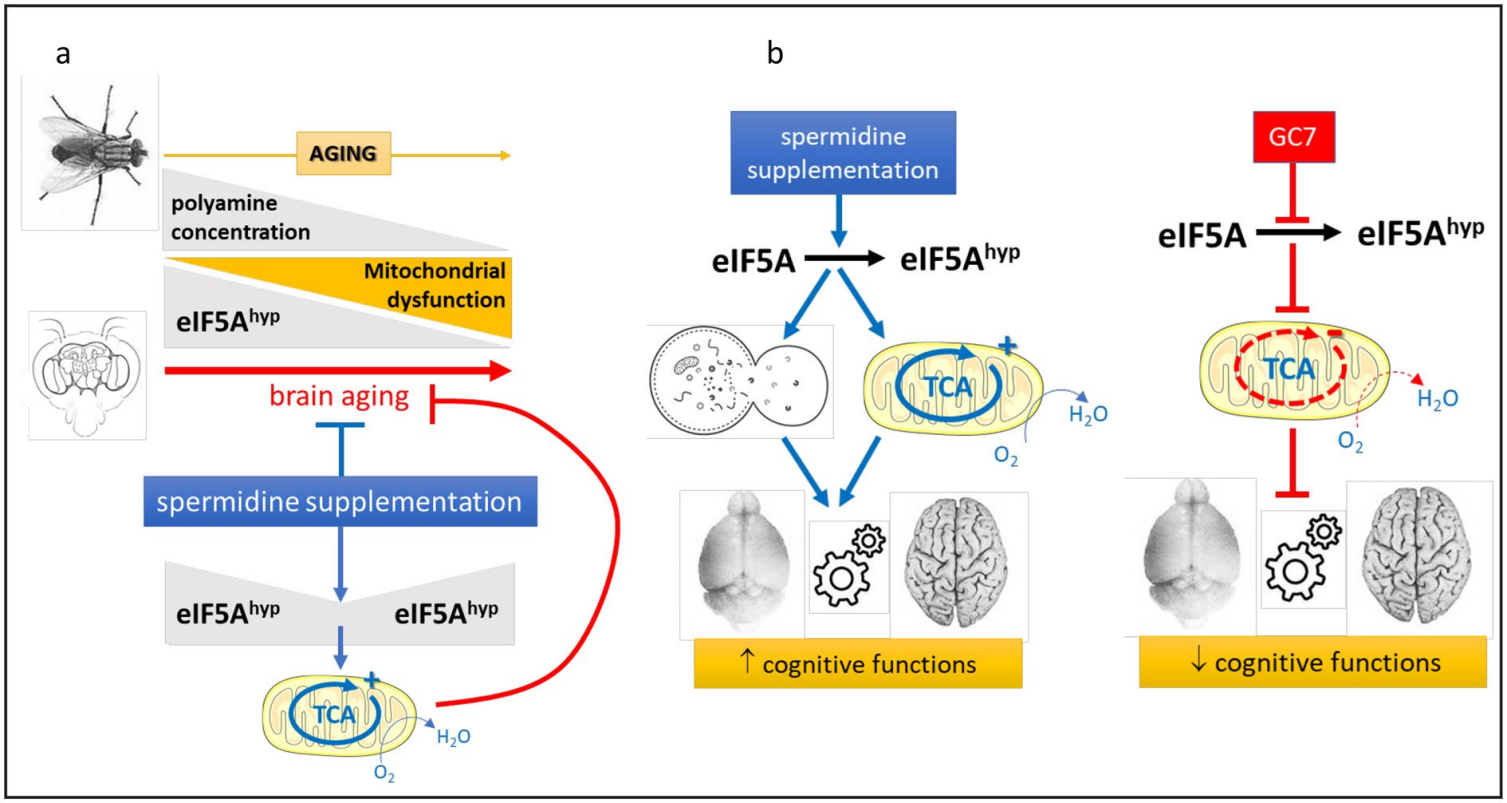
Role of eIF5A in brain aging. Hypusination of eIF5A represents a link between spermidine and its protective effect against age related senescence in the nervous system. **a** In fly, dietary spermidine supplementation maintains polyamine brain concentration and eIF5A hypusination to juvenile levels, which limits brain aging by preventing mitochondrial dysfunction. **b** In mammals, enhanced dietary spermidine intake improves eIF5A hypusination in aging brain which favors cognitive functions through autophagy and mitochondrial activity. Conversely, inhibition of eIF5A hypusination leads to mitochondrial dysfunction and to alteration of cognitive functions

Reduced eIF5A levels have been reported in the cortex and cerebellum of old rats (1.5 years) [131], the first evidence of a decrease in eIF5A production in mammalian tissues associated with aging. This reduction may contribute to the impairment of long-term motor memory observed during aging. Mitochondrial dysfunction is evident during aging, and mitochondria isolated from brain tissues showed age-related dysfunction [132]. Interestingly, food supplements fortified with polyamine and spermidine could have a beneficial effect in delaying aging and increasing longevity [133]. Gupta et al. [134] reported that the concentrations of polyamines (spermidine, putrescine) decreased in aging fruit flies, and was accompanied by a reduced performance of memorization. Simple feeding with spermidine not only restored the juvenile polyamine level, but also suppressed age-related memory disorders and preserved the ability to move [135]. In addition, efficient hypusination has been shown to be decisive for the protection of spermidine-mediated mitochondrial functionality [136]. The results obtained with the central nervous system of Drosophila provide evidence that spermidine promotes mitochondrial respiration in the course of brain aging and that brain eIF5A hypusination declines with aging (Fig. 5).

In addition, the weakening of eIF5A hypusination significantly influenced mitochondrial functionality and the protein composition in Drosophila [136] but also in mammals [84, 137]. Inhibition of eIF5A hypusination nullified a spectrum of age-related effects of spermidine on mitochondrial functionality, locomotion, and memory. Hypusination obviously has anti-aging effects on the nervous system, be it in flies, mice or humans. Therefore, the maintenance of both mitochondrial and autophagic functions is essential to improve cognition through spermidine supplementation [138]. Finally, there is a close correlation between dietary spermidine intake and eIF5A status with a reduced risk for cognitive impairment in humans [137]. In addition, it has also been shown that a higher spermidine intake is associated with lower mortality in humans [139, 140] but the total or partial involvement of hypusinated eIF5A in this effect remains to be clarified. This relationship was established in aged mice in which dietary supplementation of spermidine increased eIF5A hypusination levels in hippocampi while eIF5A protein levels remained unaffected overall [137].

While analysing the role of eIF5A in neuronal development, Kar et al. [141] engineered mice in which *Eif5a* or *Dhps* were specifically and conditionally deleted in the brain both in a regional and temporal manner. For this purpose, they used the Cre recombinase technique using *Emx1* or *CamK2a* promoters to drive the recombinase expression. In *Emx1-Cre* mice eIf5A and DHPS expression are abrogated from E9.5 throughout postnatal life in the developing rostral brain and hippocampus. In *CamK2a-Cre* mice eIF5A and DHPS are postnatally down regulated in the hippocampal cornu ammonis (CA1) neurons about 15 to 21 days after birth. The observed phenotypes showed that these animals exhibited huge alterations in spatial and contextual learning together with a strong memory deficiency. The authors attributed these observed defects in brain development or cognitive functions to induced translational errors due to a deficiency in hypusinated eIF5A.

## Spinal cord injury

The main reason for the poor prognosis of spinal cord injury (SCI) lies in neuronal loss and the limited ability of axons to regenerate after injury. Although limited, innate regenerative mechanisms exist. During spontaneous restoration of motor function after spinal cord injury, eIF5A1 and RhoGDIα (Rho GDP dissociation inhibitor alpha, a negative regulator of RHO GTPase) are up-regulated, which leads to increased neuronal survival and axonal regeneration. Down-regulation of these proteins inhibits neuroplasticity after spinal cord transection, while their over-expression rescues these protective effects [142]. eIF5A1 participates in this model by increasing both the number of neurons and neurite length, and RhoGDIα is required for eIF5A1 to function. Thus, the eIF5A pathway could represent a new target for patients with SCI.

## Immune senescence

A major hallmark of aging is the failure of the adaptive immune response, which impairs both resistance to infections and the effectiveness of vaccination in the elderly. This age-related decline has been demonstrated by Chen et al. [143] by a decrease in endogenous spermidine levels and that the decrease in the lymphocyte response could be reversed by supplementation with spermidine. The anti-aging effect of spermidine is attributed to the induction of autophagy, since a genetic change in autophagy in yeasts, worms, and flies counteracts these positive effects [144]. In agreement with this hypothesis, CD8+ T cells from aged mice and humans show reduced autophagy, and the up-regulation of autophagy in these cells restores their immune responses back to a level similar to that seen in “young” cells [145]. The relationship between spermidine, autophagy and eIF5A was published by Zhang et al. [146, 147] who reported that the control of the transcription factor EB (TFEB), a master gene for lysosomal biogenesis that controls the expression of autophagy and lysosome genes, is mediated by eIF5A hypusination. It should be noted that TFEB is a short-lived factor that contains one and two triproline motifs in mice and humans respectively, and that the mutation of this motif makes the expression of TFEB less dependent on hypusinated eIF5A while its function is not altered [146]. Finally, a decreased spermidine level in senescent B lymphocytes leads to impairment of the hypusinated eIF5A-TFEB-autophagy axis and leads to the loss of antibody responses of these cells. The same kind of results have been obtained with T lymphocytes [148].

**Fig. 6:**
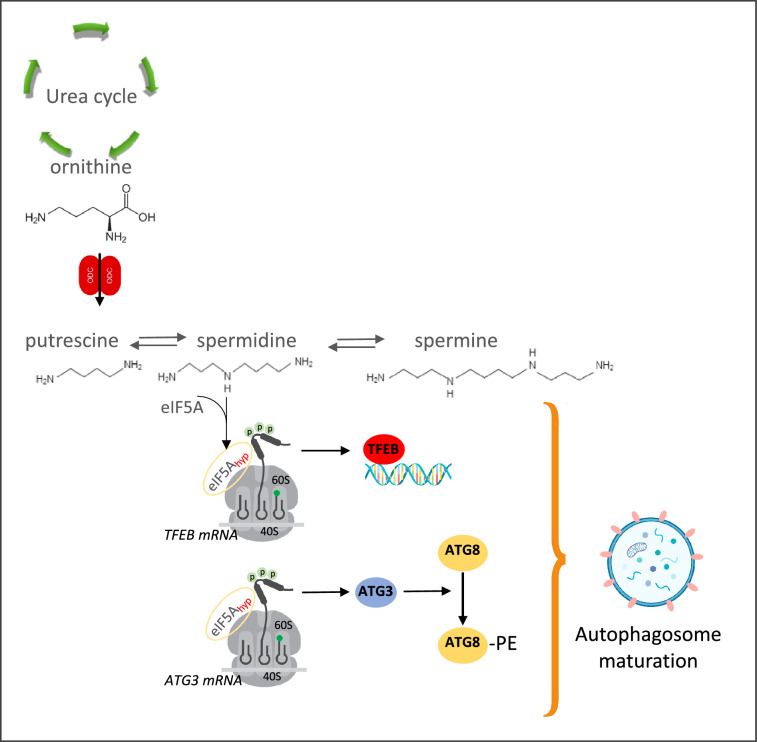
Autophagy control by eIF5A. The hypusination of eIF5A is a prerequisite for the translation of TFEB and ATG3, two tri-prolines containing proteins essential in the autophagy pathway. (1) TFEB: the member of the basic helix-loop-helix leucine-zipper family of transcription factors, is a master regulator of autophagic flux via inducing lysosome biogenesis and promoting autophagosome formation as well as its fusion with lysosome. (2) Atg3: an E2-like enzyme that catalyzes the conjugation of Atg8 and phosphatidylethanolamine (PE). The Atg8-PE conjugate is essential for the maturation of autophagosomes

TFEB is not the only protein the regulation of which by eIF5A influences autophagy. High-throughput siRNA screening identified eIF5A as a positive effector of autophagy [149], the depletion of which disrupts LC3B lipidation and inhibits the formation of autophagosomes. The autophagy-related protein ATG3 (a protein with a tripeptide motif), which has been identified as the direct translation target of eIF5A, appears to be responsible for this lipidation of LC3B. ATG3 is an E2-like conjugation enzyme that catalyzes the conjugation of ATG8 and phosphatidylethanolamine, a process essential in autophagosomes maturation (Fig. 6) [150]. Through its relationship with spermidine eIF5A appears to be a key protein in immune senescence since spermidine controls immune senescence due to a decrease in autophagy [151]. Moreover, the roles of spermidine and eIF5A and their possibility to improve immune response is of particular importance when we consider vaccine immunogenicity in older humans [148].

## Ischemia tolerance

**Fig. 7:**
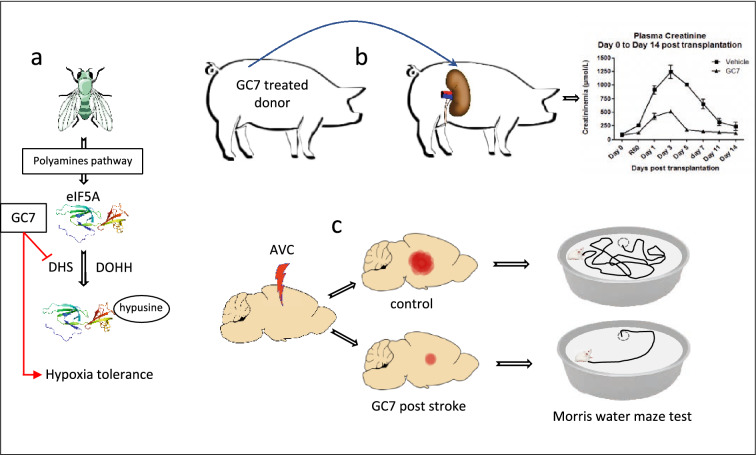
Role of eIF5A in ischemia tolerance, application to kidney transplantation and stroke. **a** Identification of the polyamine pathway and the hypusination of eIF5A as a pharmacological target in hypoxia tolerance. **b** Application of the concept in kidney transplantation in which the kidney's donor has been pretreated with GC7. The beneficial effect of GC7 is shown by the drastic decrease of the plasma creatinine level. **c** Application of the concept to the mouse model of transient cerebral focal ischemia. Mouse were treated post-stroke with GC7. The improvement of motor and cognitive behavior post-stroke was revealed by the classical Morris water maze test in which GC7 treated mice have found the immerged platform more rapidly than control mice

The first evidence that eIF5A may be involved in tolerance to ischemia was reported by Vigne and Frelin [152] who analysed the survival rate of drosophila fed various diets and exposed to hypoxia. They found that hypoxic tolerance was dramatically altered when fed a protein or amino acid enriched diet, while a diet of sucrose was beneficial. They also showed that blocking the rate limiting enzyme ornithine decarboxylase (ODC) and the polyamine pathway was protective, and ultimately demonstrated that inhibition of DHPS alone by GC7 was sufficient to induce the hypoxic tolerance (Fig. 7a). Thereafter, and because of the highly conserved expression of eIF5A between species, the possibility of a link between inhibition of eIF5A hypusination and cellular resistance to hypoxia/anoxia was investigated in renal cells originating from the proximal tubule. Data reported that treatment of these cells with GC7 induced tolerance to anoxia through a metabolic shift toward glycolysis as well as mitochondrial remodelling and led to down-regulated expression and activity of respiratory chain complexes, features characteristic of mitochondrial silencing. Treatment with GC7 also attenuated anoxia-induced generation of ROS in renal cells and in normoxic conditions, decreased their mitochondrial oxygen consumption rate [79]. In addition, intra-peritoneal injection of GC7, applied to a pathophysiological model of ischemia in rats, significantly reduced kidney levels of hypusinated eIF5A and protected against kidney damage induced by ischemia perfusion [79].

In the context of organ transplantation (a “clinical model” of ischemia–reperfusion injury), using a preclinical porcine brain death donation model, Giraud et al. [153] reported that treatment with GC7 at the beginning of the 4 h-donor management significantly improved kidney outcome during the 90-day follow-up after the transplantation. Biopsies analysis showed that GC7 increased expression of mitochondrial protective peroxisome proliferator-activated receptor-gamma coactivator-1-alpha (PGC1α) and antioxidant proteins (superoxyde-dismutase-2, heme oxygenase-1, nuclear factor [erythroid-derived 2]-like 2 [NRF2], and sirtuins). Moreover, at the end of cold storage, GC7 treatment induced an increase of NRF2 and PGC1α mRNA and a better mitochondrial integrity/homeostasis with a decrease in dynamin-related protein-1 activation and an increase in mitofusin-2. Finally, in this preclinical model, GC7 treatment was shown to be protective for kidneys against brain death-induced injuries during donor management and subsequently appeared to preserve antioxidant defenses and mitochondria homeostasis [153] (Fig. 7b).

The potential for targeting eIF5A hypusination in stroke was also recently investigated by Bourourou et al. [154]. The authors reported that GC7 induces neuroprotection against oxygen–glucose deprivation (OGD), which is associated with the preservation of mitochondrial function. They expanded these data and examined whether the administration of GC7 was able to reduce the infarct volume and functional deficits in an in vivo transient focal cerebral ischemia model in mice and they reported that post-treatment with GC7 significantly improved mouse performance, highlighting the beneficial effects of GC7 on cognitive and motor deficits after stroke (Fig. 7c).

Regarding ischemia–reperfusion, Seko et al. [155] identified a post-translationally modified eIF5A that is rapidly secreted by cardiac myocytes in response to hypoxia/reoxygenation and then acts as an autocrine-inducing apoptosis factor. The modification corresponds to a tyrosine sulfation at residue 69 that occurs in the *trans*-Golgi and that allows its secretion. An interesting result is that treatment with neutralizing anti-eIF5A mAbs significantly suppressed the hypoxia/reoxygenation induced ERK1/2 activation in cardiac myocytes and hypoxia/reoxygenation induced cytosolic release of cytochrome *c* and caspase-3 activation. Together, these changes resulted in significant suppression of apoptosis induced by hypoxia/reoxygenation. The same team reported later that this secreted form of eIF5A could mediate hypoxia/reoxygenation-induced apoptosis in neurons in vitro. This team also performed in vivo experiments and showed that the intraventricular administration of neutralizing anti-eIF5A mAbs reduced the volume of cerebral infarction after brain I/R injury [156]. In this case how the secreted form of eIF5A triggers the apoptotic signaling pathway remains to be clarified. All these recent results open up a new area of research in which targeting eIF5A could preserve cells from I/R either in pre-treatment for organ transplantation or in post-treatment following stroke.

## Mendelian disorders

These genetic disorders are mainly caused by changes or alterations in a single gene or are due to the abnormalities in the genome. Venous thromboembolism (VTE) is one of the main causes of human morbidity and from a genetic point of view many of the causal mechanisms involved in VTE have not yet been deciphered. Using a genome-wide association study (GWAS) that collected data from 18 studies Lindström [157] performed a transcriptome-wide association study of VTE in conjunction with colocalization analysis to assess the likelihood that the expression of genes in the vicinity of identified GWAS signals mediates the VTE associations described in earlier GWAS [158]. They found new VTE-associated loci, including Chr17.p13.1 in which the rs12450494 variant is intragenic, located 2.4 kb upstream of *eIF5A* and was associated only with VTE. Localisation analyses provided evidence that the rs12450494 VTE signal is mediated by the expression of eIF5A in liver and blood. Therefore, this meta-analysis provided evidences that eIF5A is related to VTE from a genomic point of view and could possibly be used as a predictive marker.

**Fig. 8:**
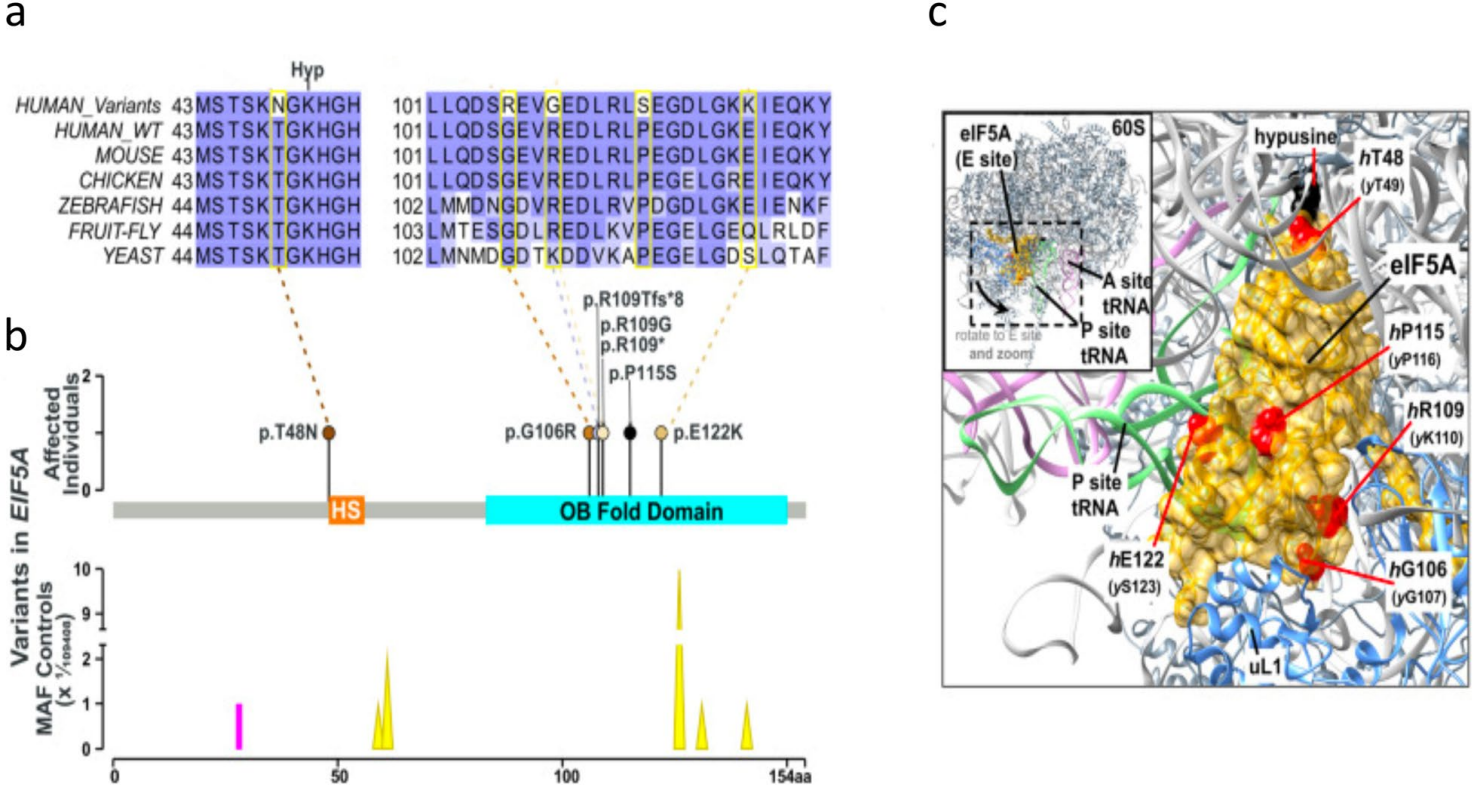
Implication of eIF5A in mendelian disorders. **a** Evolutionary conservation of residues affected by variants (delimited by yellow rectangles) is shown in five species. **b** The EIF5A variants are located in the functional sites and domains of eIF5A. **c** In silico modelling of missense variants (red spheres) supports their deleterious nature due to their proximity with the binding E-site of the 60S ribosomal subunit with adjacent P-site (green) and A-site tRNAs (pink) (Modified from Faundes et al. [159])

Very recently Faundes et al. [159] have defined a previously undescribed, potentially treatable Mendelian disease in humans that is caused by eIF5A mutations. Expression of heterozygous variants of *eIF5A* could lead to variable combinations of developmental retardation and intellectual disability such as microcephaly, micrognathy, congenital malformations and dysmorphism. Analysis of the missense variants identified in patients (Fig. 8) showed that they are located in one of the most constrained coding regions of the human genome, and that they affect the residues exposed on the surface, which are evolutionarily highly conserved. The data reported by these authors indicated that *eIF5A* variants reduced eIF5A-ribosome interaction and impaired the synthesis of proteins containing polyproline residues. Interestingly, they also showed that supplementing with spermidine the growth medium of yeasts expressing these variants could partially rescue impaired eIF5A function. This could be explained by the fact that eIF5A and spermidine can replace each other on the ribosomal P-site [160, 161]. These important data demonstrate the role of eIF5A in brain and craniofacial development, which is in part dependent on polyproline protein synthesis.

## Conclusion

eIF5A is an intriguing protein the physiological role and pathophysiological implications of which remain to be finalized. While its role has been extensively studied in cancer, its implication in normal or pathologic tissues needs to be clarified. This is particularly obvious since, depending on the protocols and tissues used, results could appear contradictory, emphasizing the major and complex role in the regulation of this protein. When considering the physiological role of eIF5A it is important to distinguish between the data into chronic inactivation, which lead to a lethal phenotype during development, and into acute pharmacological inactivation or over-expression of the protein once development is achieved. It is currently not clear whether the inhibition of the hypusination pathway alone has a similar effect as a complete depletion of eIF5A suggesting a possible function of the non-hypusinated form of eIF5A. For example, in pre-clinical approaches acute pharmacological inactivation of eIF5A hypusination is able to curb the deleterious effects of ischemic stress whereas chronic genic inactivation in other cell models can promote cell death [162]. To date, this Janus effect (when a unique treatment can lead to opposite effects depending on certain conditions) has not been really considered at its fair value. To reinforce this point of view, recent data support the fact that short, punctual and reversible inhibition of eIF5A hypusination is highly relevant in the main ischemic injuries such as transplantation and stroke and that this breakthrough should open up a new pharmacological strategy for ischemic diseases. From both a genetic and epigenetic point of view and depending on the rapid development of a meta-analysis of thousands of patients, we can expect that new relationships between eIF5A and various human diseases will be revealed. From a clinical point of view, the pharmacological targeting of eIF5A was initially considered to be a means to inhibit cell proliferation and thus as a potential treatment for cancer. However, there is now growing evidence that eIF5A and its pharmacological modulation may be envisaged in future clinical trials for other pathologies. The latest published data demonstrate clearly the substantial beneficial effect on an animal model of kidney transplantation that might be extended to other organs such as the heart, liver, or any other organs or clinical protocols involving ischemia. On other hand, the beneficial effects observed in mouse models of stroke deserve to be developed at a human level considering the time frame of treatment of stroke and the fact that inhibiting eIF5A remains effective even 2 h after the brain injury, limiting the ischemic lesions and improving recovery of function. Due to its protective role in inflammation and in macrophage polarization in obesity-induced diabetes eIF5A could be also considered as a potential factor in the improvement of glucose tolerance. Finally, and from an immunological point of view when concerning the fight against immuno-senescence the polyamines pathway and the pair spermidine/eIF5A deserve to be considered as a potential means to improve the efficacy of vaccines in the elderly. This is currently of importance at the present time to combat coronavirus disease-19 (COVID-19). To this aim, why not consider the antiviral properties of eIF5A inhibition in this field? This hypothesis is reinforced by the recent study of Gassen et al. [163] who showed that SARS-CoV-2 limits autophagy signaling and blocks autophagic flux and that this process can be reversed by exogenous administration of spermine and spermidine that are at the basis of eIF5A activation (the role of which in autophagy has been demonstrated), the AKT1 inhibitor MK-2206, and the anthelmintic drug nicosamide. They demonstrated in fine that these compounds are able to inhibit SARS-CoV2 propagation in vitro.

Finally, future genetic and physiological studies will probably reveal the specific proteins that are under the translational control of eIF5A and those primarily involved in these physiological processes.
